# Clinical and radiological differences between patients with probable cerebral amyloid angiopathy and mixed cerebral microbleeds

**DOI:** 10.1007/s00415-020-10038-8

**Published:** 2020-07-08

**Authors:** Ulf R. Jensen-Kondering, Caroline Weiler, Patrick Langguth, Naomi Larsen, Charlotte Flüh, Gregor Kuhlenbäumer, Olav Jansen, Nils G. Margraf

**Affiliations:** 1 Department of Radiology and Neuroradiology, University of Schleswig-Holstein, Campus Kiel, Arnold-Heller-Str. 3, Haus D, 24105 Kiel, Germany; 2 Department of Neurology, University of Schleswig-Holstein, Campus Kiel, Arnold-Heller-Str. 3, Haus D, 24105 Kiel, Germany; 3 Department of Neurosurgery, University of Schleswig-Holstein, Campus Kiel, Arnold-Heller-Str. 3, Haus D, 24105 Kiel, Germany

**Keywords:** Cerebral amyloid angiopathy, Modified Boston criteria, Microbleed, Hypertensive angiopathy, SWI

## Abstract

**Background:**

The key imaging features of cerebral amyloid angiopathy (CAA) are lobar, cortical, or cortico-subcortical microbleeds, macrohaemorrhages and cortical superficial siderosis (cSS). In contrast, hypertensive angiopathy is characterized by (micro) haemorrhages in the basal ganglia, thalami, periventricular white matter or the brain stem. Another distinct form of haemorrhagic microangiopathy is mixed cerebral microbleeds (mixed CMB) with features of both CAA and hypertensive angiopathy. The distinction between the two entities (CAA and mixed CMB) is clinically relevant because the risk of haemorrhage and stroke should be well balanced if oral anticoagulation is indicated in CAA patients. We aimed to comprehensively compare these two entities.

**Methods:**

Patients with probable CAA according to the modified Boston criteria and mixed CMB without macrohaemorrhage were retrospectively identified from our database. Comprehensive comparison regarding clinical and radiological parameters was performed between the two cohorts.

**Results:**

Patients with CAA were older (78 ± 8 vs. 74 ± 9 years, *p* = 0.036) and had a higher prevalence of cSS (19% vs. 4%, *p* = 0.027) but a lower prevalence of lacunes (73% vs. 50%, *p* = 0.018) and deep lacunes (23% vs. 51%, *p* = 0.0003) compared to patients with mixed CMB. Logistic regression revealed an association between the presence of deep lacunes and mixed CMB. The other collected parameters did not reveal a significant difference between the two groups.

**Conclusions:**

CAA and mixed CMB demonstrate radiological differences in the absence of macrohaemorrhages. However, more clinically available biomarkers are needed to elucidate the contribution of CAA and hypertensive angiopathy in mixed CMB patients.

**Electronic supplementary material:**

The online version of this article (10.1007/s00415-020-10038-8) contains supplementary material, which is available to authorized users.

## Introduction

The key pathological feature of cerebral amyloid angiopathy (CAA) is amyloid deposition in small cerebral parenchymal and leptomeningeal vessels [1]. In vivo diagnosis is made with the modified Boston criteria which incorporate imaging and clinical items [2]. Its hallmarks are lobar, cortical, or cortico-subcortical microbleeds [3, 4] or macrohaemorrhages and cortical superficial siderosis (cSS). Another distinct form of haemorrhagic microangiopathy is mixed cerebral microbleeds (mixed CMB). Mixed CMB is characterized by additional deep (micro)haemorrhages in the basal ganglia, thalami, periventricular white matter or the brain stem whose presence preclude the diagnosis of CAA [4]. It is unclear whether mixed CMB is a mixed form of CAA with the additional feature of hypertensive haemorrhages or a severe form of hypertensive microangiopathy [5, 6]. A variant or severe form of CAA seems unlikely since deep structures are relatively spared from amyloid deposition [7, 8]. Previous studies on patients with CAA and mixed CMB with macrohaemorrhages could detect clinico-radiological differences driven by vascular risk factors [6].

We hypothesized that differences between these two groups are already present in the absence of macrohaemorrhages. In this study, we aimed to determine the clinical and radiological differences between these two groups without macrohaemorrhages.

## Materials and methods

The study was approved by the local ethics committee (Ethikkommission der Medizinischen Fakultät der Christian-Albrechts-Universität zu Kiel). Patients consented to the retrospective use of acquired data at hospital admission. If consent for usage was not given, only anonymized data were used.

**Fig. 1:**
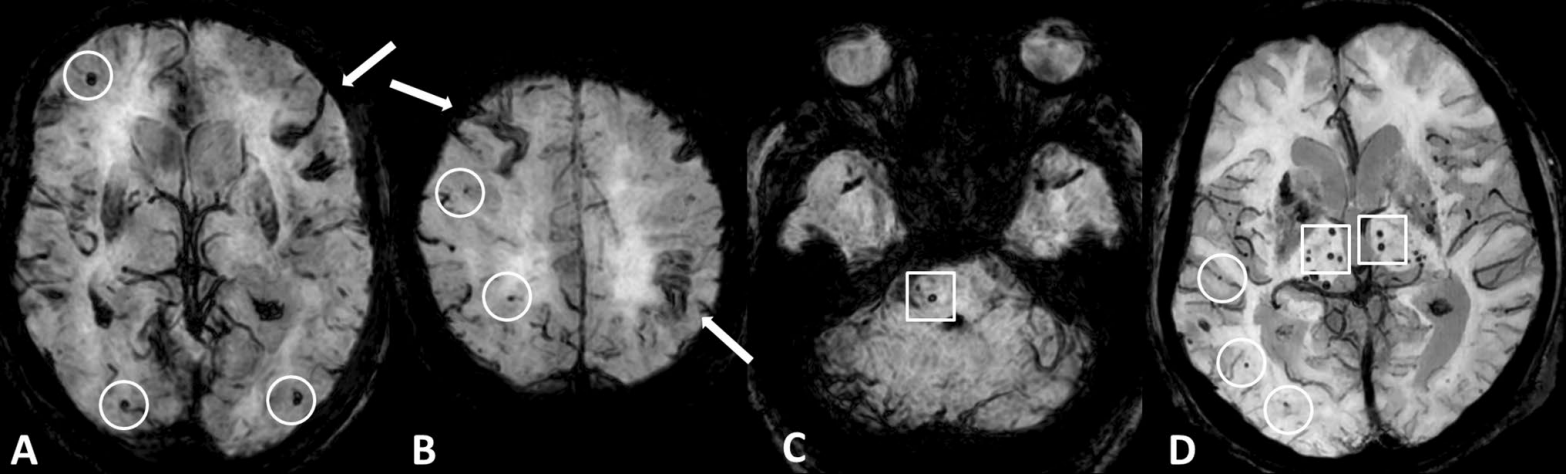
**a**, **b** Susceptibility-based MRI in a patient with probable CAA. Cerebral microbleeds in lobar location (circles) and cortical superficial siderosis (arrows). **c**, **d** Susceptibility-based MRI in a patient with mixed CMB. Cerebral microbleeds in lobar location (circles) and additional deep microbleeds (boxes) in the brain stem (**c**) and thalami (**d**)

We retrospectively included patients based on a search in our image database. Patients were included from 2014–2019 and there were no restrictions concerning the requesting department or clinic. Patients were included in the CAA group if they had an MRI at our institution including a susceptibility based sequence (e.g., SWI, SWIp), for sequence parameters see Supplemental Table 1) and fulfilled at least the criteria for probable CAA (possible CAA excluded) according to the modified Boston criteria [2]. Patients were included in the mixed CMB group if they fulfilled the before mentioned criteria for CAA but had additional CMB in the basal ganglia, thalami, periventricular white matter and/or brain stem (Fig. 1).

Clinical data of the patients were taken from the patients’ charts by a medical student with a Bachelor of Science in psychology (CW) blinded to the results of the radiological analysis. Specifically, the following parameters were extracted: age, sex, reason for MRI, previous ischemic stroke, arterial hypertension, number of antihypertensive drugs, diabetes mellitus, insulin dependency, hyperlipidaemia, creatinine (mg/dl), LDL (mg/dl), oral anticoagulation or platelet inhibitors, smoking habits, dementia (other than AD).

Any of the following was an exclusion criterion: Antecedent relevant head trauma, brain irradiation, blood dyscrasia or coagulopathy, warfarin overdose (INR > 3), central nervous system tumor, vascular malformation or cerebral vasculitis as per the modified Boston criteria [2], clinical or radiological signs of previous or acute ICH, surgery with heart–lung-machine [9] and Alzheimer’s disease (according to criteria of the National Institute on Aging-Alzheimer’s Association (NIA-AA) [10] or criteria of the International Working Group (IWG-2) [11] based on retrospective chart review and inclusion of all available data (neuropsychological testing, imaging, CSF, etc.) of current, previous and following hospital stays. Any dementia not fulfilling these criteria was rated as “dementia”.

Distinction between CAA and mixed CMB was made by one board certified radiologist (UJK) based on susceptibility-based images and other available imaging material. A second rater (NL) blinded to all other results also dichotomized patients into CAA and mixed CMB to calculate interrater agreement. Additionally, the number of superficial microbleeds was dichotomized into high (> 10) and low (≤ 10) [12] by two raters in consensus (UJK and NGM, board certified neurologist). A microbleed was defined as a homogenous small (2–10 mm) round or ovoid signal void on susceptibility based images not traceable through adjacent slices to distinguish them from vessels as defined in the STRIVE criteria [13].

Total white matter lesion (WML) volume was delineated on FLAIRw images (for sequence parameters see Supplemental Table 1) as previously described [14] by one board certified radiologist (UJK) in a blinded manner. In short, a manual outline of the white matter lesions was drawn in MRIcron (https://www.people.cas.sc.edu/rorden/mricron/index.html) and defined as a region of interest. A second region of interest was defined by choosing an individually adjusted intensity threshold. Total WML volume was defined as the intersection of the two regions of interest.

**Table 1:** Clinical and radiological characteristics of patients with CAA and mixed CMB

Characteristics	CAA (*n* = 52)	Mixed CMB (*n* = 44)	*p* value
Age, mean (SD) years	78 (8)	74 (9)	**0.036**^a^
Female sex, *n* (%)	23 (44)	19 (43)	0.918^b^
Reason for MRI			
Suspected Stroke, *n* (%)	33 (62)^e^	24 (53)^f^	0.358^b^
Seizure, *n* (%)	5 (9)^e^	4 (9)^f^	1^c^
Dementia, *n* (%)	2 (4)^e^	6 (13)^f^	0.247^c^
Other, *n* (%)	13 (25)^e^	12 (26)^f^	0.907^b^
Clinical event to MRI, median, (range) days	3 (0–15)^n=43^	3 (0–17)^*n*=37^	0.315^d^
Acute ischemic stroke, *n* (%)	22 (42)	18 (41)	0.889^b^
Previous ischemic stroke, *n* (%)	22 (42)	22 (49)	0.516^b^
Anticoagulation or platelet inhibition, *n* (%)	30 (61)^*n*=49^	30 (66)	0.483^b^
Diabetes mellitus, *n* (%)	12 (24)^*n*=51^	10 (23)	0.926^b^
Insulin dependency, *n* (%)	4 (33)	5 (50)	0.665^c^
Arterial hypertension, *n* (%)	38 (75)^*n*=51^	39 (89)	**0.079**^b^
Number of antihypertensive drugs, median (range)	2 (0–6)	2 (0–6)	0.736^d^
Hypercholesterinemia, *n* (%)	18 (38)^*n*=48^	19 (43)	0.578^b^
LDL, mean (SD) mg/dl	2.86 (0.875)^*n*=35^	3.00 (1.25)^*n*=35^	0.587^a^
Statin medication, *n* (%)	21 (44)^*n*=48^	13 (30)^*n*=44^	0.158^b^
Creatinine, mean (SD) mg/dl	91 (27.73)^n=45^	89.51 (27.76)^n=41^	0.805^a^
Smoking, *n* (%)	13 (34)^n=38^	7 (22)^n=32^	0.255^b^
Dementia, *n* (%)	13 (25)	14 (32)	0.459^b^
WML pattern subcortical, *n* (%)	39 (75)	30 (68)	0.459^b^
WML pattern peri BG, *n* (%)	9 (17)	10 (23)	0.386^b^
WML pattern posterior, *n* (%)	48 (92)	37 (84)	0.223^b^
WML pattern frontal, *n* (%)	36 (69)	35 (80)	0.226^b^
WMH volume, mean (SD) ml	14 (11)^n=51^	18 (19)^n=43^	0.138^a^
BG EPVS high degree (score > 20), *n* (%)	36 (69)	30 (68)	0.912^b^
CSO EPVS high degree (score > 20), *n* (%)	29 (56)	22 (50)	0.572^b^
Lacunes ≥ 1, *n* (%)	26 (50)	32 (73)	**0.023**^b^
Rate of lobar lacunes (%)	73	69	0.81^b^
Rate of deep lacunes (%)	23	51	**0.0003**^**b**^
High CMB count (> 10), *n* (%)	25 (48)	30 (67)	**0.065**^b^
Presence of cSS, *n* (%)	10 (19)	2 (4)	**0.027**^b^

*SD* standard deviation, *MRI* magnetic resonance imaging, *LDL* low density lipoprotein, *WML* white matter lesions, *BG* basal ganglia, *EPVS* enlarged perivascular spaces, *CSO* centrum semiovale, *CMB* cerebral microbleeds, *cSS* cortical superficial siderosis

^a^*t* test

^b^Chi-square test

^c^Fisher’s exact test

^d^Mann–Whitney *U* test

One (^e^) and two (^f^) patients with more than one reason for MRI

Bold values indicate *p* < 0.1

Four mutually not exclusive patterns of white matter damage were identified on FLAIRw or T2w images by one board certified radiologist (PL) blinded to the results of the other analysis: (1) subcortical white matter spots (*n* > 10) in a subcortical location, (2) white matter spots following the outline of the basal ganglia, (3) white matter patches (> 5 mm) anterior of the frontal horn and (4) or posterior of the posterior horn of the lateral ventricles [15]. Additionally, dichotomized scores were given for the presence or absence of lacunes (≥ 1) and high number (> 20) of enlarged perivascular spaces (EPVS) on the level of the basal ganglia and the centrum semiovale. A lacune was defined as a > 3 mm round or ovoid fluid filled cavity with a hyperintense rim on FLAIRw images whereas an EPVS was defined as a > 1 and < 3 mm linear fluid filled space following the course of vessels and traceable through several adjacent slices without a hyperintense rim on FLAIRw images as defined in the STRIVE criteria [13, 16, 17]. A second rater (UJK) categorized lacunes as previously described [18] into “lobar” if they were located in the centrum semiovale or frontal, parietal, temporal, occipital or the insular lobe and “deep” if they were located in the thalamus, the internal or external capsule or in the basal ganglia in a blinded manner.

One board certified radiologist and one board certified neurologist (NGM and UJK) in consensus identified location and extent of cSS using the susceptibility-based datasets which were manually transferred into a predefined T1w set of images adapted from a publicly available brain template (ch2 template [19]) covering the whole brain. cSS was defined as a curvilinear hypointense rim on susceptibility based images following the cortex not visible on FLAIRw images to distinguish them from acute subarachnoid haemorrhage [2].

R (version 3.5.0, R Foundation for Statistical Computing, Vienna, Austria) and StatView (Version 5.0.1, SAS Institute Inc., Cary, NC, USA) were used for statistical calculations. Mean, standard deviation, median, ranges and proportions are displayed for variables as appropriate. Comparisons were made with a two-sided *t* test for normally distributed continuous variables, Mann–Whitney *U* test for non-normally distributed or ordinal scale variables, for categorical variables Chi-square test or Fisher’s exact test (expected frequencies < 5). If group differences for a variable reached statistical significance, a logistic regression was performed. A *p* value of < 0.05 was considered statistically significant.

## Results

96 patients (CAA = 52, mixed CMB = 44) were included. Interrater agreement for the diagnostic categorization CAA vs. mixed CMB was near perfect (*κ* = 0.84). Patients’ characteristics are listed in Table 1.

In the CAA group (*n* = 13), the reason for MRI “Other” was as follows: work-up of retinal aneurysms (*n* = 1), follow-up of imaging in other hospital (*n* = 1), incidental finding in study MRI (*n *= 1) and screening MRI for spine surgery (*n* = 1), screening for cerebral metastasis (*n* = 3), unspecific neurologic symptoms, like headache, dizziness, difficulty walking, etc. (*n* = 6), and in the mixed CMB group (*n* = 12): follow-up of imaging in other hospital (*n* = 1), screening for cerebral metastasis (*n* = 1), unspecific neurologic symptoms (*n* = 10). Patients screened for cerebral metastasis had no signs of intracranial malignant manifestation neither in the MRI of interest nor in follow-up imaging. 22 out of 33 and 18 out of 24 patients with suspected stroke were finally diagnosed with an acute ischemic stroke in the CAA and mixed CMB group respectively. Two patients in the CAA group and one patient in the mixed CMB group initially suspected to have ischemic stroke were finally diagnosed with “Transient Focal Neurological Episode” (TFNE).

Patients with CAA were older (78 ± 8 vs. 74 ± 9 years, *p* = 0.036) and had a higher prevalence of cSS (19% vs. 4%, *p* = 0.027) but a lower prevalence of lacunes (73% vs 50%, *p* = 0.018) compared to patients with mixed CMB. Presence of deep lacunes was more common in the mixed CMB group (23% vs. 51%, *p* = 0.0003), but there was a similar rate of lobar lacunes (73% vs 69%, *p* = 0.81) in the CAA and the mixed CMB group.

**Fig. 2:**
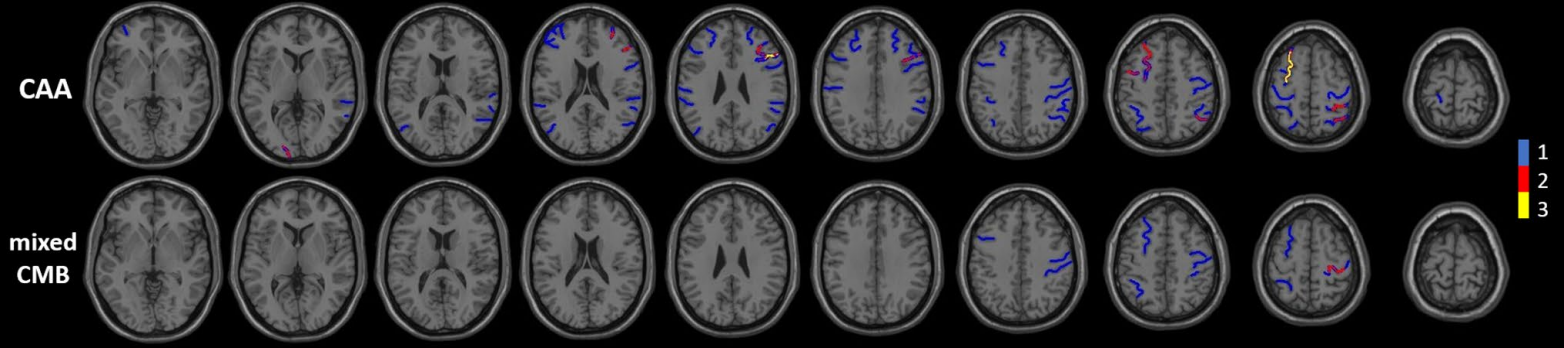
Comprehensive depiction of the location, extent and cumulative quantity of cortical superficial siderosis in CAA patients (*n* = 10, 19%) and mixed CMB patients (*n* = 2, 4%). No cSS was detected below the level of the basal ganglia

Location and extent of cSS are displayed in Fig. 2. No further significant correlation was found between the location and extent of cSS and other radiological and clinical parameters. It is however, worth noting that only supratentorial convexity cSS was present.

For the variables age, presence of lacunes, presence of deep lacunes and presence of cSS only the presence of deep lacunes was significantly associated with the diagnosis mixed CMB [*β* = 0.41, *p* = 0.0002, OR (95% CI) = 1.51 (1.22–1.85)]. The other collected parameters did not reveal a significant difference between the two groups. However, there was a trend (*p* < 0.10) toward high microbleed count (*p* = 0.065) and the presence of arterial hypertension (89% vs. 67%, *p* = 0.071) in patients with mixed CMB.

## Discussion

The main finding from this study is that CAA and mixed CMB have a different clinical and radiological profile in the absence of macrohaemorrhages, although there is a large overlap between the two cohorts.

This is the first comparison study between patients with CAA and mixed CMB without macrohaemorrhage. In a cohort of patients with sustained macrohaemorrhage, Pasi et al. could demonstrate differences mainly driven by vascular risk factors [6]. They also found an increased rate of lacunes and arterial hypertension in patients with mixed CMB, while CAA patients displayed a higher rate of cSS. Further, they could demonstrate a higher rate of diabetes mellitus, left ventricular hypertrophia, increased serum creatinine and typical white matter damage in patients with mixed CMB. Smith et al. compared patients with CAA and hypertensive vasculopathy and macrohaemorrhages, but could also not detangle the contribution of both in mixed CMB patients [5]. They concluded that some of these patients might have CAA. In our study the only variables with significantly differences between CAA and mixed CMB were the presence of cSS which was more common in the CAA group and of lacunes which were more frequent in mixed CMB patients. Additionally, deep lacunes were a predictor of mixed CMB resembling the results of Pasi et al. [18] in patients with mixed location haemorrhage who also displayed significantly more deep lacunes. This might be explained by the fact that patients in our study in the absence of macrohaemorrhages are in an earlier stage of hypertensive disease and other possible severe end organ damage is not yet present. Second, the presence of arterial hypertension and high CMB count at least show a trend towards a significant difference. Third, the number of patients is considerably smaller compared to the above mentioned studies maybe mitigating a possible difference between the groups. Studies on patients with CAA and mixed CMB and macrohaemorrhages in European [20] and Asian [21] populations found that CMB is mainly driven by hypertensive vasculopathy although a subgroup of these patients might have coexisting or dominant CAA. Further studies on the natural history of mixed CMB should shed more light on the early stages of the disease. This is of particular interest to identify patients who might benefit from risk factor modification.

Previous studies demonstrated that lobar micro- and macrohaemorrhages might be caused by arterial hypertension which classically is thought to cause deep cerebral haemorrhages. Kim et al. demonstrated that amyloid burden and hypertensive small vessel disease have synergistic effects on the progression of lobar microbleeds [22]. This might be reflected in our study by the statistical trend for a higher number of patients with > 10 microbleeds in the mixed CMB group. The rate of CMB in the general population was reported to be 8.8% of which 64% were lobar, 19% were deep and 34% mixed [23]. Deep and mixed CMB were both associated with arterial hypertension. Yakushiji et al. found a high proportion of mixed CMB in patients with intracranial haemorrhage. These patients showed the severest white matter damage [24]. However, no strict definition of CAA or mixed CMB such as the Boston criteria [25] or the modified Boston criteria [2] was used in these two reports.

Further, in our study the diagnostic value of convexity cortical superficial siderosis is reemphasized. Only supratentorial convexity cSS was present (Fig. 2) making it distinct from infratentorial and spinal cSS with the classical clinical triad of sensorimotor hearing loss, cerebellar symptoms and pyramidal signs [26]. Although not a common finding, cSS seems highly specific for CAA in the absence of other explanations (95% in this cohort). This is also appreciated by the modified Boston Criteria. By additionally incorporating cSS to the established criteria for haemorrhage distribution it was possible to increase the sensitivity of the Boston criteria [25] from 89.5 to 94.7% without compromising specificity [2].

The distinction between the two entities (CAA and mixed CMB) is clinically relevant because oral anticoagulation in patients with CAA should be carefully considered under certain circumstances and requires a differentiated look at the pattern and quantity of haemorrhage distribution [27]. On the other hand withholding oral anticoagulation from patients who may have CAA and a clear indication for oral anticoagulation is equally critical and the risk of haemorrhage and stroke should be well balanced [28].

An alternative stroke prevention therapy in patients with atrial fibrillation and a high bleeding risk might be the use of a left atrial appendage occlusion [29]. The treatment dilemma between bleeding risk due to CAA and necessity of anticoagulation, e.g., due to arterial fibrillation has a growing relevance because with an aging society stroke patients will be older and therefore the risk of CAA in these cohorts is increasing. Also, mixed CMB is increasingly recognized as a prevalent condition which makes the distinction clinically more meaningful. However, at present we cannot answer whether mixed CMB is a mixture of CAA and hypertensive microangiopathy or a maximum form of hypertensive microangiopathy. Consequently, other diagnostic parameters are needed.

The CSF profile might be used to identify CAA patients. Reduced Aβ40 has been reported in patients with CAA when compared to healthy controls consistent with the pathological correlate of preferential amyloid Aβ40 deposition in cerebral vessel [30, 31]. However, more investigations are needed.

Retinal abnormalities in the vasculature have been described in CAA patients [32]. However, data on this condition are scarce and no correlation with current clinical or radiological severity scales has been performed yet.

PET imaging with amyloid specific tracers provides insights into the presence and distribution of amyloid deposition [21, 33] but in clinical routine PET imaging with tracers such as ^11^C-PiB (Pittsburgh Compound B) are not readily available.

Notable strengths of our study are the strict application of the modified Boston Criteria for the definition of the CAA group and conversely for the mixed CMB group. We additionally excluded patients with previous surgery with heart–lung-machine which can result in a radiological picture mimicking CAA or mixed CMB [9]. Further, radiological inclusion was based on a susceptibility-based sequence in all patients. Susceptibility-based sequences are known to depict considerably more microbleeds and cortical superficial siderosis compared to GRE T2* sequences [34]. Furthermore, we comprehensively collected clinical data on the patients of two cohorts.

A limitation of this study is its retrospective nature. Although we cannot exclude selection bias due to clinical presentation, symptom severity or delay from symptom onset to MRI, no such difference was seen in the group analysis. Compared to other prospectively maintained databases like the “Massachusetts General Hospital Haemorrhagic Stroke Research Program” our cohort is relatively small. However, it is unique when regarding the patients’ disease progression with none of the patient having experienced symptomatic intracranial haemorrhage. Another drawback is the lack of pathological confirmation in our cohort. We are very well aware that other pathologies might explain the clinic-radiological picture in a part of the patients. However, we did not include the category “Possible CAA” according to the modified Boston Criteria and used additional exclusion criteria to keep diagnostic certainty as high as possible.

## Conclusion

CAA and mixed CMB show a different clinical and radiological profile in the absence of macrohaemorrhages. However, to differentiate these two entities more clinically available diagnostic parameters are necessary.

## Electronic supplementary material

Below is the link to the electronic supplementary material.

Supplementary file1 (DOCX 12 kb)
