# Supplementary material for: Clinical and radiological differences between patients with probable cerebral amyloid angiopathy and mixed cerebral microbleeds
Source: J Neurol. 2020 Jul 8;267(12):3602–8. doi: 10.1007/s00415-020-10038-8 (PMC7674181; doi:10.1007/s00415-020-10038-8)
Supplement: Supplementary file 1 — Supplementary file1 (DOCX 12 kb) [file 415_2020_10038_MOESM1_ESM.docx]

**Supplemental Table 1:** Imaging details

**SIEMENS Aera, 1.5 T**

Sequence parameters:

Susceptibility based imaging (SWI): repetition time (TR) 49 ms, echo time (TE) 40 ms, field of view (FOV) 75x75 mm, flip angle (FA) 15°, slice thickness (SL) 2 mm, (n=16, CAA=6, MLH=10, p=0.143^a^)

Fluid attenuated inversion recovery (dark fluid): repetition time (TR) 9000 ms, echo time (TE) 87 ms, inversion time (TI) 2500, field of view (FOV) 100x100 mm, flip angle (FA) 150°, slice thickness (SL) 3.5 mm, (n=15, CAA=5, MLH=10, p=0.076^a^)

**Philips Achieva, 1.5 T**

Susceptibility based imaging (Veno BOLD): repetition time (TR) 34.44 ms, echo time (TE) 49.445 ms, field of view (FOV) 86.54x86.54 mm, flip angle (FA) 15°, slice thickness (SL) 2.2 mm, (n=49, CAA=31, MLH=18, p=0.07^a^)

Fluid attenuated inversion recovery (FLAIR): repetition time (TR) 10000 ms, echo time (TE) 125 ms, inversion time (TI) 2800, field of view (FOV) 95.31x95.31 mm, flip angle (FA) 90°, slice thickness (SL) 4 mm, (n=49, CAA=31, MLH=18, p=0.067^a^)

**Philips Achieva, 3 T**

Susceptibility based imaging (SWIp): repetition time (TR) 18.94 ms, echo time (TE) 26.866 ms, field of view (FOV) 79.69x79.69 mm, flip angle (FA) 15°, slice thickness (SL) 2 mm, (n=31, CAA=15, MLH=16, p=0.432^a^)

Fluid attenuated inversion recovery (FLAIR): repetition time (TR) 12000 ms, echo time (TE) 160 ms, inversion time (TI) 2850, field of view (FOV) 95.31x95.31 mm, flip angle (FA) 90°, slice thickness (SL) 3 mm, (n=30, CAA=15, MLH=15, p=0.477^a^)

^a^: Chi-square test
